# SARS-CoV-2 and Its Variants: The Pandemic of Unvaccinated

**DOI:** 10.3389/fmicb.2021.749634

**Published:** 2021-09-24

**Authors:** Fabrizio Angius, Giuseppe Pala, Aldo Manzin

**Affiliations:** Microbiology and Virology Unit, Department of Biomedical Sciences, University of Cagliari, Cittadella Universitaria, Cagliari, Italy

**Keywords:** SARS-CoV-2 mutants, vaccination effectiveness, pandemic control measures, COVID-19 handling, concerning variants

## Introduction

Freshly emerged Severe Acute Respiratory Syndrome Coronavirus-2 (SARS-CoV-2), related to SARS-CoV (Rangan et al., 2020; Zehender et al., 2020) and promptly identified as the causative agent for severe respiratory diseases and the potentially life-threatening syndrome COVID-19, accounts for the current pandemic with more than 4 million deaths (WHO, 2021a). As part of the *Riboviria* group, SARS-CoV-2 harbors an RNA-dependent RNA polymerase which, despite a rudimental exonuclease competence, is partially responsible for the low accuracy in its genome replication together with other non-mutual features; however, the said facets may result in an actual fitness advantage. Therefore, it is foreseeable that when most individuals are susceptible, variants may arise and become a concerning phenomenon; in fact, mutations that mainly regard the Spike (S) protein, increased infectivity, and viral fitness have recently been documented. The S protein is a fusion protein responsible for binding to specific cellular receptors and mediates membrane fusion, the earliest steps in the infection process. In the S protein, a cleavage site separates S1 domain—which harbors residues that specifically recognize and bind to receptors—from the C-terminal S2 where two heptad repeats are responsible for conformational changes and 6-helix bundle formation, leading to fusion between viral envelope and cell membrane. Particularly, S1 possesses two high mutagenic surface areas: the N-Terminal Domain (NTD) and the Receptor Binding Domain (RBD). NTD has 3 supersites (N1, N3, and N5), where all known anti-NTD antibodies bind (Winger and Caspari, 2021); RBD has 17 residues that directly tether the angiotensin-converting enzyme 2 (ACE2) expressed on target cells (i.e., endothelial and epithelial cells, myofibroblasts, enterocytes) (Hamming et al., 2004). Currently, four main variants of concern (VOCs) have been identified based on transmissibility competence and immune-escape potential (Davis et al., 2021; Funk et al., 2021; Lopez Bernal et al., 2021). In fact, among several viral factors, transmissibility represents one of the most important intrinsic features for efficient fitness, which may be affected by control measures adopted in response to the pandemic such as confinement, use of personal protective equipment, and social distancing. Moreover, immune escape is a further relevant element that may positively affect viral fitness through less sensitivity to humoral or cellular immune response. Furthermore, aspects correlated with an increased replication rate may affect viral invasion and clinical outcomes, although are yet to be clarified.

### The Pandemic Progression and Emerging Variants

**Figure 1:**
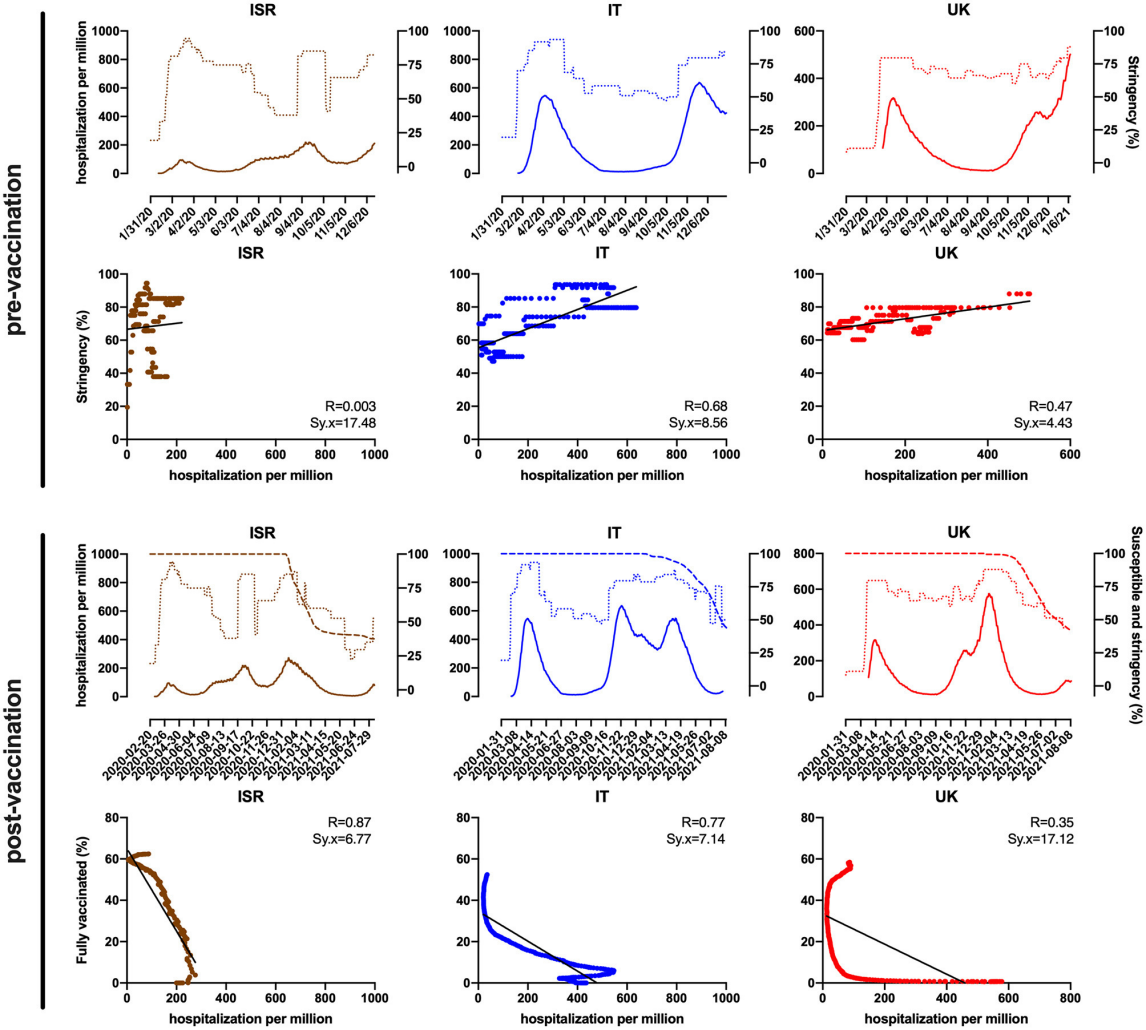
Israel (ISR), Italy (IT), and the United Kingdom (UK) trend (top) and correlation (below) of hospitalizations per million (hard line) as a function of stringency index (dotted line) between January 2020 and the beginning of mass vaccination rollout (pre-vaccination) and as a function of fully vaccinated (%) and susceptible individuals (%) defined as individuals who did not receive full vaccination (dashed line), between December 2020 and August 2021 (post-vaccination). Data were obtained and modified from the WHO COVID-19 Situation Reports and Our World in Data (Mathieu et al., 2021; WHO, 2021b).

The SARS-CoV-2 pandemic initially showed a high emergency rate aggravated by the sole available countermeasure aimed at limiting the transmission and the number of people exposed. Beyond restrictions and closures, containment and health policies embraced measures such as testing and contact tracing, short-term investments in healthcare, and vaccines. However, this action is difficult to compare between countries as it suffers from a high variability. Indeed, stringency is measurable and determined using ordinary containment and public strategies representing the most reliable index to define the progression of the pandemic. Despite the overtime variability, delay in response, and the non-cumulative effect, increasing stringency showed a remarkable drop in the number of hospitalizations and consequently in the fatality rate (Figure 1). Interestingly, when only stringency was available to limit transmission, a new variant with higher transmissibility became predominant (CDC, 2021a; Davies et al., 2021). In September 2020, the UK reported a new variant (Alpha) belonging to B.1.1.7 lineage and presenting mutations in the S that sensibly favor transmissibility (+43–90%) compared with the wild-type virus (Wuhan/A-lineage). This gain of function caused widespread occurrence of Alpha all over Europe (Canton et al., 2021), especially in countries with low-vaccination level and mostly affecting non-immunized or partially immunized people. The Alpha S protein carries four deletions and seven substitutions including D614G, which increases 2- to 8-fold the viral titer in the lungs (Korber et al., 2020) and N501Y conferring 10-fold higher affinity to ACE2 (Winger and Caspari, 2021). In addition, although full vaccination by mRNA vaccines stimulates an efficient neutralizing antibody response (84–93% against Alpha), a drop to 30–50% in protection has been reported between shots and immediately after recall, suggesting a partially protective effect (Polack et al., 2020; WHO, 2021b). Alpha acquired an additional mutation (E484K) with immune-escape potential conferring 7-fold more resistance to neutralization by sera (Edara et al., 2021). Over time, the virus continued its pressing race especially in high-density and developing countries, where inadequate control measures and social behavior gave the virus further chances to randomly acquire additional gain-of-function mutations. It is the case of the South African and Indian variants, which boast higher transmissibility and immune escape than Alpha (Lazarevic et al., 2021; Mahase, 2021). In October 2020, South Africa identified the 501Y.V2 variant (B.1.351), Beta, which possesses an S that binds more efficiently to ACE2 due to three mutations in the RBD and is endowed with higher immune escape against acquired immune response (WHO, 2021b). Beta carries the same mutations as the P.1 identified in Brazil (Gamma), including K417N that is responsible for partial immune escape and N501Y that reduces affinity of RBD-directed monoclonal antibodies (Fratev, 2021). Moreover, Beta S protein also has some deletions that prevent binding of anti-NTD monoclonal antibodies (Wall et al., 2021). Contemporarily, a new variant (B.1.617) was reported in India (Delta) with P681R altering the furin cleavage site and L452R endowing higher transmissibility (+55% vs. Alpha) and immune-escape potential than other VOCs (+1.5-fold vs. Beta, +4-fold vs. Alpha), respectively (Davis et al., 2021; Planas et al., 2021). Recently, Delta reached the UK, resulting in a remarkably increased number of cases and is currently spreading all over Europe. Delta carries many mutations with the Q1071H defining three new sub-clades (B.1.617:A, B.1.617:B, B.1.617:C) and the following sub-variants: B.1.617.1 (Kappa), B.1.617.2 (Delta), and B.1.617.3. Overall, this evidence suggests a highly dynamic evolution of B.1.617 S protein (Winger and Caspari, 2021) and very recent studies show more than 5-fold reduced *in vitro* effectiveness of vaccine-induced neutralizing antibodies against this lineage (Liu et al., 2021). Recently, a new variant B.1.617.2.1 (Delta plus) has been identified with the mutation K417N in the S known to increase the propensity of RBD to maintain an open configuration, hence chances to bind to ACE2 (CDC, 2021b; Fratev, 2021). Noteworthy, C-37 (Lambda), indicated as a variant of interest first reported in Peru and endowed with higher infectivity compared with Alpha and Gamma (Acevedo et al., 2021), is on the rise in South America and also detected outside the Americas.

### The Vaccination Issues

In December 2020, after an inconceivable effort, several vaccines were developed and approved for urgent use; thereafter, a huge mass vaccination campaign started in many developed countries. On-label indications state that vaccine-induced immunity is maximized when the vaccination is complete, whereas vaccine effectiveness between doses is yet to be clarified, although it is believed to be higher than that of naive (Paris et al., inpress). To clarify the vaccination weight in the pandemic, we consider the post-vaccination period in UK and Italy as representative of European countries, using Israel as a comparison model because of its moderate size, the incisive government actions to limit infections, and that 53% of the population received full vaccination very shortly (Dagan et al., 2021). Figure 1 clearly shows the strong negative correlation between hospitalizations as a function of percentage of fully vaccinated in Israel (R = 0.87; Sx.y = 6.77) and Italy as well (R = 0.77; Sx.y = 7.14), meaning that vaccination represents a highly efficient tool to limit the disease, although a limited effect of stringency cannot be completely excluded. In contrast, the correlation shows to be weaker for the UK (R = 0.35; Sx.y = 17.12) likely due to different vaccination strategies, emergence of variants, and demographics. Nevertheless, despite highly effective vaccines being steadily set up and made available, some have recently shown reduced effectiveness against one or more variants. In fact, the virus has shown a certain benefit in its evolutionary potential due to the unpaired vaccination rate between countries and to aspects strictly linked to the viral advantage (see Supplementary Tables). In particular, the uneven worldwide vaccination rate (a meager 16% of the world's population is fully vaccinated) (Mathieu et al., 2021) highlights the urge of a fair mass vaccination to significantly reduce the global number of susceptible individuals to limit viral transmission and circulation; indeed, Delta and related variants are becoming dominant in different countries (CDC, 2021c; Edara et al., 2021). Recently, it has been reported that incomplete vaccination does not grant sufficient immune response, hence protection, making those exposed likely susceptible to infection (Lopez Bernal et al., 2021; Canaday et al., inpress). This is more evident in the UK, compared with Israel and Italy, where the government primarily opted for a wide semi-vaccination strategy rather than full vaccination, in the effort to provide the highest percentage of population with limited immunization. This proved to be strategic as initially the number of cases drastically dropped over time until the highly transmissible Delta variant, which had more chances to become dominant, spread (Campbell et al., 2021; Hagen, 2021; Pascarella et al., 2021). Contrarily, Italy went through specific government policies such as targeted lockdowns with regional variation and different health strategies based on full vaccination, despite a consistent hesitancy in vaccination adherence. Initially, Italy has been one of the most damaged countries in Europe in terms of number of cases and fatality rate, but government countermeasures and mass full vaccination led to resume Europe's average. Moreover, due to its effectiveness, several governments kept a moderate stringency even after the vaccination began, granting a mutual effect—although non-synergic—among the plethora of unvaccinated.

## Discussion and Closing Remarks

The pandemic progression in a country also depends on other relevant constant factors that are distinctive of a specific population, which include geographical (i.e., transport interconnection, insularity) and demographic features (i.e., density, average age, fragility, genetics). Therefore, in countries with disadvantageous features, containment and healthcare policies such as vaccination, testing/sequencing, and tracing (governance) play a fundamental role. The cooperative and simultaneous action of all governances reveals to be the most effective to contain this exceptional event. In fact, although stringency alone can limit the number of exposed people, it does not directly affect fatality rates or viral advantages. On the other hand, vaccines have shown to directly affect the fatality rate and partially the viral advantage, which is strictly dependent on the replication rate. Furthermore, other healthcare strategies such as tracing and testing may give information about the pandemic progression and be highly effective when coupled with containment and prevention measures.

Early in the pandemic, all governments were forced to use stringency as a unique possible countermeasure, leading to a significant control of the virus advantage, although with a high fatality rate. In EU countries with peculiar demographics such as Italy, which are very heterogeneous among regions, governance has been adapted locally to contain the spread of infection. In December 2020, EU began a mass vaccination for healthcare workers and fragile people to continue with elderly and younger individuals, and recently some specific cohorts are receiving a heterologous vaccination that seems to confer a stronger immune response (Barros-Martins et al., 2021). Locally, Sardinia significantly differs among Italian regions in intrinsic demographics such as high average age (46.8 years) and the presence of roughly 19% of fragile individuals. In addition, the rate of susceptible subjects among the elderly is rather high (24.28%), consisting in more than 125,000 people (IStat, 2021). Genome variability is a common feature among viruses, but in a pandemic scenario is heightened and supported by the large number of susceptible subjects—mostly unvaccinated in this part of pandemic—offering the virus more chances to mutate and prospective selection of variants, in particular those boasting an increased transmissibility and immune-escape potential. In this context, to limit the emergency, the fatality rate and viral advantage are the major issues to categorically shrink. To date, several governances are available, and their effective management has already shown a high impact in fatality rate reduction. Still, it remains necessary to administrate an even worldwide mass vaccination to limit the viral advantage as long and as widely as possible, making vaccines available particularly in developing or high-density populated countries, or where preventive measures are lacking and they fail to limit the virus' ability to replicate and spread, therefore promoting the pandemic's progression.

## Author Contributions

FA, GP, and AM: substantial contribution to the conception, analysis, and interpretation of data. FA, GP, and AM: drafting the work and revising it critically for important intellectual content. All authors provide approval for publication of the content and agree to be accountable for all aspects of the work in ensuring that questions related to the accuracy or integrity of any part of the work are appropriately investigated and resolved.

## Funding

The present work has been supported by Grant No. 2017M8R7N9/PRIN 2017 from the Italian Ministry for University and Research (MIUR).

## Conflict of Interest

The authors declare that the research was conducted in the absence of any commercial or financial relationships that could be construed as a potential conflict of interest.

## Publisher's Note

All claims expressed in this article are solely those of the authors and do not necessarily represent those of their affiliated organizations, or those of the publisher, the editors and the reviewers. Any product that may be evaluated in this article, or claim that may be made by its manufacturer, is not guaranteed or endorsed by the publisher.
