# Supplementary material for: SARS-CoV-2 and Its Variants: The Pandemic of Unvaccinated
Source: Front Microbiol. 2021 Sep 24;12:749634. doi: 10.3389/fmicb.2021.749634 (PMC8497702; doi:10.3389/fmicb.2021.749634)
Supplement: Supplementary file 1 [file Table_1.docx]

Supplementary Material

**Supplementary Table 1.** Transmissibility of SARS-CoV-2 main Variants of Concern (VOCs), and effectiveness of vaccines against these variants. WT: Wild-type; ND: Not Determined.

|  | **Transmissibility** | **Viral vector vaccines**  **Efficacy** | **mRNA-based vaccines**  **efficacy** | **Viral sub-unit vaccines**  **Efficacy** | **Inactivated virus vaccines**  **Efficacy** | **Attenuated virus vaccines**  **efficacy** |
| --- | --- | --- | --- | --- | --- | --- |
| **ALPHA (B.1.1.7)** | **Increased**  43 - 90% > WT | **70 - 80%** | **> 80%** | **> 80%** | **> 80%** | **On trial** |
| **BETA**  **(B.1.351)** | **Increased**  50% > WT | **< 70%** | **70 - 80%** | **< 70%** | **70 - 80%** | **On trial** |
| **GAMMA**  **(P.1)** | **Increased**  2.6% > WT | **70 - 80%** | **> 80%** | **ND** | **> 90%** | **On trial** |
| **DELTA (B.1.617.2)** | **Increased**  55% > Alpha | **> 80%** | **70 - 80%** | **ND** | **ND** | **On trial** |
