# Supplementary material for: SARS-CoV-2 and Its Variants: The Pandemic of Unvaccinated
Source: Front Microbiol. 2021 Sep 24;12:749634. doi: 10.3389/fmicb.2021.749634 (PMC8497702; doi:10.3389/fmicb.2021.749634)
Supplement: Supplementary file 2 [file Table_2.docx]

Supplementary Material

**Supplementary Table 2.** Amino acid substitutions and deletions (X) in SARS-CoV-2 main Variants of Concerns (VOCs). Shared substitutions of particular interest (in italic) are: D614G and N501Y, conferring increased transmissibility, and E484K conferring enhanced immune-escape potential.

| **Amino acid position** | **WILD-TYPE (A.1.1)** | **ALPHA**  **(B.1.1.7)** | **BETA**  **(B.1.351)** | **GAMMA**  **(P.1)** | **DELTA (B.1.617.2)** |
| --- | --- | --- | --- | --- | --- |
| 18 | L | - | - | F | - |
| 19 | T | - | - | - | R |
| 20 | T | - | - | N | - |
| 26 | P | - | - | S | - |
| 69 | H | X | - | - | - |
| 70 | V | X | - | - | - |
| 80 | D | - | A | - | - |
| 138 | D | - | - | Y | - |
| 144 | Y | X | - | - | - |
| 145 | Y | X | - | - | - |
| 156 | E | - | - | - | G |
| 157 | F | - | - | - | X |
| 158 | R | - | - | - | X |
| 190 | R | - | - | S | - |
| 215 | D | - | G | - | - |
| 241 | L | - | X | - | - |
| 243 | A | - | X | - | - |
| 417 | K | - | N | T | - |
| 452 | L | - | - | - | R |
| 478 | T | - | - | - | K |
| 484 | E | - | *K* | *K* | - |
| 501 | N | *Y* | *Y* | *Y* | - |
| 570 | A | D | - | - | - |
| 614 | D | *G* | *G* | *G* | *G* |
| 655 | H | - | - | Y | - |
| 681 | P | H | - | - | R |
| 701 | A | - | V | - | - |
| 716 | T | I | - | - | - |
| 950 | D | - | - | - | N |
| 982 | S | A | - | - | - |
| 1027 | T | - | - | I | - |
| 1118 | D | H | - | - | - |
| 1176 | V | - | - | F | - |
